# Effect of shear stress on iPSC-derived human brain microvascular endothelial cells (dhBMECs)

**DOI:** 10.1186/s12987-017-0068-z

**Published:** 2017-08-04

**Authors:** Jackson G. DeStefano, Zinnia S. Xu, Ashley J. Williams, Nahom Yimam, Peter C. Searson

**Affiliations:** 1 Institute for Nanobiotechnology, Johns Hopkins University, 100 Croft Hall, 3400 North Charles Street, Baltimore, MD 21218 USA; 2 Department of Materials Science and Engineering, Johns Hopkins University, Baltimore, MD 21218 USA; 3 Department of Biomedical Engineering, Johns Hopkins University, 720 Rutland Avenue, Baltimore, MD 21205 USA

**Keywords:** Shear stress, Brain microvascular endothelial cells (BMECs), Human endothelial cell line, Blood–brain barrier, Endothelial turnover, Cell morphology, Cell motility, Stem cells

## Abstract

**Background:**

The endothelial cells that form the lumen of capillaries and microvessels are an important component of the blood–brain barrier. Cell phenotype is regulated by transducing a range of biomechanical and biochemical signals in the local microenvironment. Here we report on the role of shear stress in modulating the morphology, motility, proliferation, apoptosis, and protein and gene expression, of confluent monolayers of human brain microvascular endothelial cells derived from induced pluripotent stem cells.

**Methods:**

To assess the response of derived human brain microvascular endothelial cells (dhBMECs) to shear stress, confluent monolayers were formed in a microfluidic device. Monolayers were subjected to a shear stress of 4 or 12 dyne cm^−2^ for 40 h. Static conditions were used as the control. Live cell imaging was used to assess cell morphology, cell speed, persistence, and the rates of proliferation and apoptosis as a function of time. In addition, immunofluorescence imaging and protein and gene expression analysis of key markers of the blood–brain barrier were performed.

**Results:**

Human brain microvascular endothelial cells exhibit a unique phenotype in response to shear stress compared to static conditions: (1) they do not elongate and align, (2) the rates of proliferation and apoptosis decrease significantly, (3) the mean displacement of individual cells within the monolayer over time is significantly decreased, (4) there is no cytoskeletal reorganization or formation of stress fibers within the cell, and (5) there is no change in expression levels of key blood–brain barrier markers.

**Conclusions:**

The characteristic response of dhBMECs to shear stress is significantly different from human and animal-derived endothelial cells from other tissues, suggesting that this unique phenotype that may be important in maintenance of the blood–brain barrier. The implications of this work are that: (1) in confluent monolayers of dhBMECs, tight junctions are formed under static conditions, (2) the formation of tight junctions decreases cell motility and prevents any morphological transitions, (3) flow serves to increase the contact area between cells, resulting in very low cell displacement in the monolayer, (4) since tight junctions are already formed under static conditions, increasing the contact area between cells does not cause upregulation in protein and gene expression of BBB markers, and (5) the increase in contact area induced by flow makes barrier function more robust.

**Electronic supplementary material:**

The online version of this article (doi:10.1186/s12987-017-0068-z) contains supplementary material, which is available to authorized users.

## Background

The blood–brain barrier (BBB) is a dynamic interface that separates the brain from the circulatory system and protects the central nervous system from potentially harmful chemicals while regulating transport of essential nutrients [1, 2]. Endothelial cells in the brain are highly specialized with tight junctions that effectively block paracellular transport and an array of transporters and efflux pumps that control entry into the brain. A reliable source of human, brain-specific cells has been a major barrier to developing BBB models [3], however, stem cell technology provides a solution to this problem [4–6]. Human iPSC-derived BMECs (dhBMECs) show expression and localization of tight junction proteins, very high transendothelial electrical resistance (TEER > 2000 Ω cm^2^), low permeability, and polarized expression of P-gp efflux pumps [4–6].

Previous studies have been performed under static conditions, and hence the goal of this study is to assess the influence of shear stress on dhBMECs in confluent monolayers. Shear stress can play a profound role on endothelial morphology and function, regulating signaling and transport between blood and surrounding tissues [7–9]. In straight sections of large vessels under laminar flow, endothelial cells (ECs) are elongated and aligned in the direction of flow [10–13]. In 2D cell culture, confluent monolayers of many ECs elongate and align in the direction of flow [7, 8, 10–21], recapitulating EC morphology in larger vessels. As a result of the similarity in morphology in large vessels and in 2D monolayers, elongation and alignment under shear stress is thought to be a hallmark of ECs [10, 11, 14, 16, 19, 22–24]. In previous work we have shown that immortalized brain microvascular endothelial cells do not exhibit this characteristic elongation and alignment in response to shear stress [19] or in response to curvature [25], suggesting that hBMECs have a unique phenotype.

Here we assess the morphology, cell motility, rates of proliferation and apoptosis, and protein and gene expression of dhBMECs in 2D confluent monolayers under shear stress in comparison to static conditions. We show that dhBMECs exhibit a unique phenotype in response to shear stress: (1) they do not elongate and align, (2) the rates of proliferation and apoptosis decrease, (3) the mean displacement of individual cells within the monolayer over time is significantly decreased, (4) there is no cytoskeletal reorganization or formation of stress fibers within the cell, and (5) there is no change in expression levels of key blood–brain barrier markers. This phenotype is significantly different from human and animal derived endothelial cells from other tissues, indicating that dhBMEC have a unique phenotype that may be important in maintenance of the blood–brain barrier.

## Methods

### Cell culture

Human brain microvascular endothelial cells (dhBMECs) were differentiated from the BC1 human induced pluripotent cell (hiPSC) line (provided by Dr. Linzhao Cheng, Johns Hopkins University). Details of the differentiation and characterization of the hBMECs have been reported elsewhere [4]. Briefly, all cells were cultured in T25 and T75 flasks (Falcon, Tewksbury, MA, USA) with daily media changes. BC1-hiPSCs were cultured in colonies on 40 µg mL^−1^ Matrigel-treated tissue culture dishes (Corning, Tewksbury, MA, USA) and maintained in TeSR-E8 media, changed daily (Stem Cell Technologies, Vancouver, Canada). BC1-hiPSCs were passaged using StemPro^®^ Accutase^®^ solution (Life Technologies, Waltham, MA, USA). 10 µM ROCK inhibitor Y27632 (ATCC, Manassas, VA, USA) was included in the TeSR-E8 culture media for the first 24 h after passaging. After culture for 3–4 days in TeSR-E8, the media was switched to unconditioned media without basic fibroblast growth factor (bFGF) (UM/F- media) to induce the differentiation. The cells were maintained in this media for 6 days with daily media replacement. The UM/F- media is composed of DMEM/F12 (Life Technologies) supplemented with 20% KnockOut Serum Replacement (Life Technologies), 1% non-essential amino acids (Life Technologies), 0.5% l-glutamine (Sigma-Aldrich, St. Louis, MO, USA), and 0.84 µM beta-mercaptoethanol (Life Technologies). The media was then switched to endothelial cell media (EC) for 2 days to promote growth of the endothelial cells. The EC media is composed of endothelial cell serum-free media (Life Technologies), supplemented with 1% human platelet poor derived serum (Sigma-Aldrich), 20 ng mL^−1^ bFGF (R&D Systems), and 10 µM all-trans retinoic acid (Sigma-Aldrich). After 2 days in EC media, the cells were sub-cultured into the microfluidic devices.

### Microfluidic platform

**Fig. 1:**
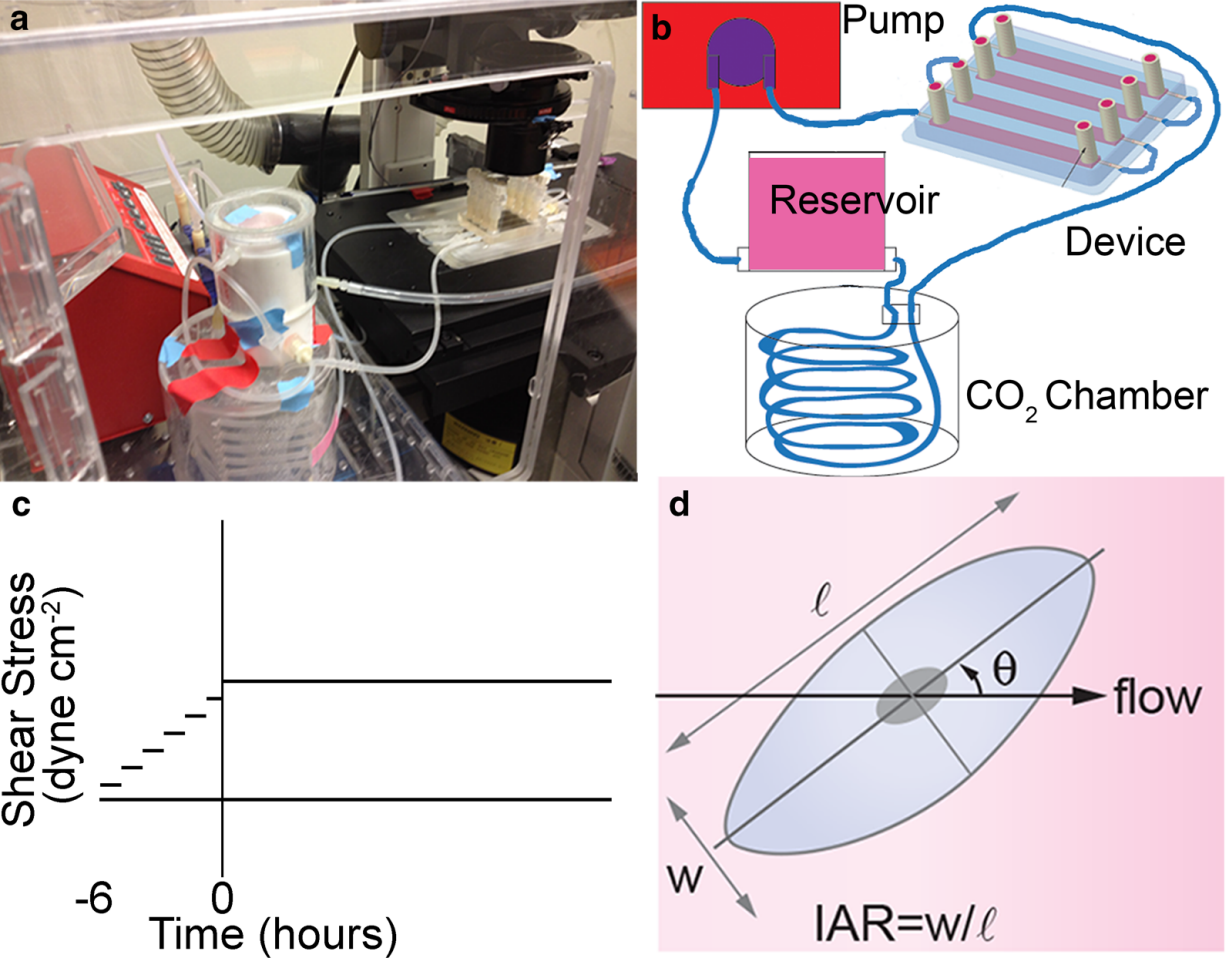
Microfluidic platform. **a** Image of a device and flow loop in the live cell chamber on the microscope. **b** Schematic illustration of the flow loop including: a peristaltic pump, a media reservoir, a CO_2_ chamber, and the microfluidic device. **c** Profile of the applied shear stress during experiments. On forming a confluent monolayer, the shear stress is increased stepwise over 6 h to a maximum flow rate of 10 mL min^−1^ for the duration of the experiment. **d** Schematic illustration of an endothelial cell illustrating the morphological parameters of interest, *l* length of long axis, *w* length of short axis, *IAR* inverse aspect ratio (w/*l*), *θ* orientation angle of long axis with respect to flow direction

The microfluidic device and flow loop were fabricated as reported previously (Fig. 1a, b) [19]. Briefly, polydimethylsiloxane (PDMS, Sylgard 184 silicon elastomer kit, Dow Corning, Midland, MI, USA) was cast in an aluminum mold to create four rectangular channels with different heights to allow simultaneous measurements at different shear stresses. The PDMS channels were plasma bonded to a 50 mm × 75 mm glass microscope slide (Corning). The flow loop included a custom-machined Teflon media reservoir connected via 1/8″ ID silicon tubing to a peristaltic pump (NE-9000, New Era Pump Systems, Farmingdale, NY, USA) that was programmed to steadily ramp up flow and obtain final shear stresses of 4 and 12 dyne cm^−2^ in respective channels of the device. Channels under static conditions (0 dyne cm^−2^) were not connected to the flow loop.

The dhBMECs were seeded into the microfluidic devices after 48 h sub-culture. Each microfluidic device has four channels: two static (0 dyne cm^−2^) channels, a 4 dyne cm^−2^ channel, and a 12 dyne cm^−2^ channel. All channels were coated with a 1:1 mixture of 50 µg mL^−1^ fibronectin (Sigma-Aldrich) and 100 µg mL^−1^ collagen IV (Sigma-Aldrich) for 12 h prior to cell seeding. A confluent T25 of sub-cultured dhBMECs was washed three times with PBS without Ca^2+^ and Mg^2+^, followed by a prolonged wash, approximately 7 min, with TrypLE™ Express (Life Technologies) at 37 °C to gently dissociate the cells from the culture flask. Two to three million cells were collected and then spun down to a pellet and the excess media aspirated away. 400 µL of EC media was then added to the pellet and mixed using a pipette such that all the cells from one T25 are suspended in 400 µL. Each channel was seeded with 100 µL of cell suspension corresponding to approximately 500,000 cells per channel. Additional media was added to fill each channel (54 µL in the 4 dyne cm^−2^ channel and 122 µL in the 12 dyne cm^−2^ channel). The cell density is relatively high to ensure the formation of a confluent monolayer since non-adherent cells are washed away with the addition of media. To demonstrate that the seeding density does not play a significant role in cell behavior, experiments were also performed with 250,000 cells and 125,000 cells seeded per channel. Cells were allowed to settle and attach to the fibronectin/collagen IV coated glass slide for about 2 h at which point 1 mL of media was added to each channel to wash away cells that did not attach, and the monolayers were allowed to grow to confluence, approximately 24 h, at 37 °C and 5% CO_2_. We aimed to start experiments at an average cell area of between 800 and 1000 µm^2^. If after 24 h, the average cell area was outside this range, the experiment was not performed. For static experiments (0 dyne cm^−2^), cells were seeded using the same protocol but not connected to the flow loop.

After formation of a confluent monolayer, the microfluidic device was connected to a peristaltic pump, gas exchange chamber, and media reservoir for live-cell imaging. The channels requiring flow (4 and 12 dyne cm^−2^ channels) were connected in series via tubing to the peristaltic pump, whereas the 0 dyne cm^−2^ channels were not connected to the flow loop. The peristaltic pump was programmed to increase flow from 1.25 to 10 mL min^−1^ over 6 h. The flow rate was then maintained at 10 mL min^−1^ for 40 h unless otherwise stated. The time at which the maximum flow rate was reached (after the 6 h conditioning period) is designated as the zero time point. Experiments were performed in EC media, composed of endothelial cell serum free media (Life Technologies), supplemented with 1% human platelet poor derived serum (Sigma-Aldrich), 20 ng mL^−1^ bFGF (R&D Systems, Minneapolis, MN, USA), and 10 µM all-trans retinoic acid (Sigma-Aldrich). For cell maintenance and to avoid overgrowth and formation of mounds, media was replaced every 24 h in the static channels. To assess the role of vasomodulators on dhBMEC monolayers, some experiments were performed in EC media containing either (1) 400 µM DB-cAMP or (2) 10 µM ROCK inhibitor. The flow system was maintained at 37 °C and humidified with 5% CO_2_ for the duration of the experiments. After 6 h conditioning and 40 h under the designated shear stress, the monolayers were either immediately fixed for immunofluorescence staining or prepared for genetic or proteomic analysis.

### Live-cell imaging

To assess the response of dhBMECs to flow, confluent monolayers were imaged under static conditions (0 dyne cm^−2^) or under a shear stress of 4 or 12 dyne cm^−2^ for 40 h in a custom microfluidic device (Fig. 1). A shear stress of 4 dyne cm^−2^ is representative of the average shear stress in the venous system (typically 1–4 dyne cm^−2^) and 12 dyne cm^−2^ is representative of the average shear stress in capillaries (typically 10–20 dyne cm^−2^) [26–32].

Live-cell time lapse imaging was performed using a Nikon TE-2000U inverted microscope controlled by NIS Elements Software (Nikon, Tokyo, Japan) with a 10× Nikon Plan Fluor objective. Imaging was performed at three locations in each channel: in the center of the channel and at points 10 mm from either end of the channel. The locations were centered approximately 2 mm from either side wall, to avoid edge effects. Time lapse images were recorded for 46 h with images taken every 20 min. Autofocus adjustment was performed before each image capture to correct for any z-drift. The number of cells in each imaging region (1.5 mm × 1.2 mm) was about 2000. All experiments were performed in triplicate (three microfluidic devices with three imaging locations per device) and hence all parameters represent an average of about 18,000 cells at each time point.

### Morphological analysis

Quantitative analysis of cell morphology was performed using ImageJ (NIH, Bethesda, MD, USA) and techniques previously developed in our lab [19]. Images of the cell monolayers from time-lapse movies were imported into ImageJ and the cell borders were delineated automatically using a custom macro [19]. Morphological parameters (inverse aspect ratio, orientation angle, and cell area) of individual cells were obtained as long as more than 85% of the monolayer could be traced by this method. The automated analysis of cell monolayers from phase contrast images was validated by comparison to analysis by manually tracing cell boundaries in immunofluorescence images at the same time point [19].

### Turnover analysis

Quantitative analysis of cell proliferation and apoptosis was performed using ImageJ. Proliferation events were identified visually from cell division and the formation of daughter cells. Apoptosis and cell loss from the monolayer was apparent from pronounced cell contraction and detachment events. Both proliferation and apoptosis events are readily identified in phase contrast time-lapse images (Additional files 1, 2 and 3). Individual division and apoptosis events occur over 20–40 min spanning 1–3 frames. Proliferation and apoptosis events were identified and quantified under both static and shear flow conditions. Time-lapse videos of cell monolayers were imported as stacks of image sequences and cell division and apoptotic events counted manually every 20 min. Proliferation and apoptosis rates are reported as % h^−1^. Analysis was performed at each of the three imaging locations in respective channels to obtain the rates of cell division and apoptosis for each shear stress and media condition. To determine the net rate of change in cell number (% h^−1^), the apoptosis rate was subtracted from the division rate. Identification of apoptosis and proliferation events from phase contrast movies allows quantitative analysis of the dynamic behavior of the monolayer as a function of time [33, 34]. Furthermore, direct observation ensures that we include apoptosis events associated with cell loss and removal from the monolayer by shear flow, which may not be detected by labeling methods. To ensure that proliferation and apoptosis event counting was reproducible, analysis was performed by five different observers. Post-evaluation analysis revealed that less than 5% of the events were misidentified or not counted, and there was no statistical difference between independent analysis of the same time lapse images.

### Cell motility analysis

To assess cell motility we measured three parameters: cell speed, root mean square (RMS) displacement, and directionality. Cell speed, a measure of the average velocity of cells moving within the monolayer, is a directionless velocity with units of µm min^−1^. RMS displacement is a measure of how far a cell moves from its original position in a monolayer as a function of time. Finally, directionality is a measure of the direction of cell motion with respect to the flow direction. Quantitative analysis of cell speed was performed using OpenPIV [35] using methods reported previously [19]. Image sequences of cell monolayers from time-lapse movies were imported into OpenPIV and analyzed using particle image velocimetry (PIV). Reproducible approximations of monolayer speed were obtained between each successive image and reported over time as averages of triplicate experiments. The cell speed obtained from PIV was validated by manual tracking of individual cells (Additional file 4: Figure S1).

Root mean square displacement and directionality were quantified by manually tracking the location of the center of cell nuclei throughout an experiment. RMS displacement is quantified as the magnitude of the vector from the starting location of a cell to the current location, and is a measure of how far a cell in a confluent monolayer moves over time. The displacement is measured for at least 10 cells in each of the three imaging locations. Directionality is quantified as the change in x- or y-direction between two frames and is reported in microns. RMS displacement and directionality were obtained for at least 100 cells over three independent experiments.

### Immunofluorescence imaging

After time-lapse live-cell imaging, monolayers were immediately fixed for immunofluorescence staining and imaging. Cell monolayers were first washed twice in 1× PBS with Ca^2+^ and Mg^2+^, and fixed in 3.7% formaldehyde (Fisher Scientific Hampton, NH, USA) in PBS for 5 min. Next, the samples were washed three times with PBS and permeabilized with 0.1% Triton-X 100 (Sigma-Aldrich Aldrich). The samples were subsequently washed three times in PBS and blocked with 10% donkey serum in PBS for 1 h. The samples were then incubated with primary antibodies overnight at 4 °C. Primary antibodies include claudin-5 (Thermo Fisher Scientific, #35-2500), occludin (Thermo Fisher Scientific, #40-4700), and ZO-1 (Thermo Fisher Scientific, #40-2200). The samples were washed three times with PBS for 5 min each on a rocker. The samples were then incubated with DAPI nuclear stain (Roche Applied Science), Alexa Fluor 488 phalloidin (F-actin, Thermo Fisher Scientific), and secondary antibodies. Immunofluorescence images were taken using a Nikon Eclipse Ti-E inverted microscope controlled by NIS Elements Software (Nikon). Images were obtained from similar locations to the phase contrast images to minimize possible edge effects. Immunofluorescence images were quantified for claudin-5, occludin, and ZO-1 expression, and F-actin orientation. To assess junctional expression, cell–cell boundaries were traced using ImageJ (from one edge of the image field to the other edge three times per image) and the average pixel intensity minus the background was collected and averaged [36]. To assess F-actin orientation, FibrilTool was used to find the average orientation of the fibers within each cell [37].

### Protein analysis

Confluent monolayers of cells were lysed immediately after time-lapse imaging experiments using RIPA buffer (Sigma-Aldrich) containing protease inhibitor cocktail (Sigma-Aldrich). Samples were centrifuged at 25,000 RPM for 25 min at 4 °C, and stored at −20 °C. Western blots were performed on 4–15% pre-cast polyacrylamide gels (Bio-Rad, Hercules, CA, USA). The bands were transferred from the gels onto nitrocellulose membranes (Bio-Rad), and blocked with 5% fat-free skim milk (Bio-Rad) in TBS (Corning) with 0.05% TWEEN-20 (Sigma-Aldrich) for 1 h at room temperature. Primary antibodies (Additional file 4: Table S1) were added to the milk cocktail and incubated overnight at 4 °C. Membranes were washed three times for 5 min each with TBS with 0.05% TWEEN-20. Secondary HRP antibodies (Bio-Rad) were added to milk and incubated for 1 h at room temperature before imaging (Bio-Rad molecular imager ChemiDoc XRS+) using ImageLab 5.1 software. β-actin was used as a loading control. Western blots were performed in quadruplicate for CLDN-5 and LAT-1 and triplicate for ZO-1 using lysate from three or four independent experiments. Analysis of relative intensities of the bands was performed using imageJ. Each lane was normalized and compared against the intensity of the 0 dyne cm^−2^ lane to reduce the influence of the background.

### Gene analysis

Quantitative PCR (qPCR) was performed using an Applied Biosystems StepOnePlus Real-time PCR system to assess changes in mRNA expression in the following genes: *ABCB1*, *CDH5*, *CLDN5*, *OCLN*, *SLC2A1*, and *TJP1*, with *ACTB* and *GAPDH* as the housekeeping genes. PCR samples were prepared using the TaqMan^®^ Gene Expression Cells-to-CT™ Kit (Life Technologies). Cells were washed twice in PBS, dissociated with StemPro^®^ Accutase^®^ solution (Life Technologies) and lysed with the cells-to-CT lysing solution (Life Technologies). Fold changes were analyzed using the comparative C_T_ method (∆∆C_T_) [38] normalizing to *ACTB* and *GAPDH* expression and comparing to static conditions (0 dyne cm^−2^) as a reference.

### Statistics

To determine statistical significance, we use a two-tailed Student’s *t* test to compare two samples with unequal variances, with a p value of 0.05 being the threshold for significance (p ≤ 0.05 = *; p ≤ 0.01 = **; p ≤ 0.001 = ***).

## Results

### Morphology

**Fig. 2:**
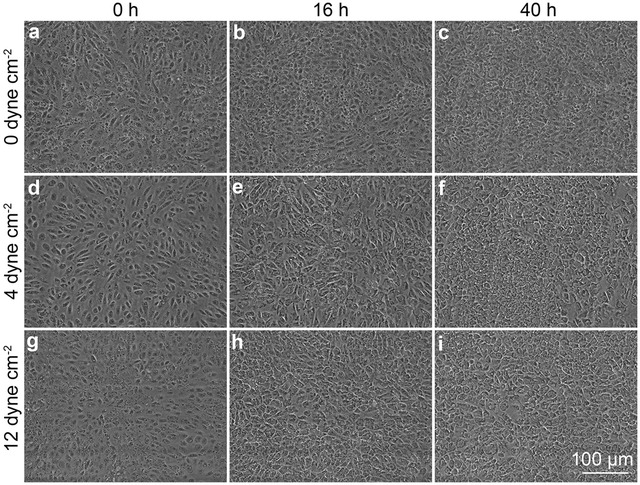
Representative phase contrast images of confluent dhBMEC monolayers at 0, 16, and 40 h. **a**–**c** Static conditions (0 dyne cm^−2^). **d**–**f** 4 dyne cm^−2^. **g**–**i** 12 dyne cm^−2^

From phase contrast images, the dhBMECs initially show a cobblestone morphology with well-defined cell nuclei and subtle cell–cell junctions under all conditions (Fig. 2). At longer times the nuclei become less well-defined and the cell–cell junctions become more distinct due to increased overlap and flattening of the cells. At higher magnification it is also evident that organelles and other intracellular vesicles become more pronounced. Despite these changes in appearance, the cells maintain their cobblestone morphology under shear stress (the average IAR and orientation angle remain the same). The key results, described below, are summarized in Table 1.

**Table 1:** Summary of steady state results from this study (dhBMECs) and previous studies (Other ECs)

dhBMEC			Other EC		
Steady state	Static	Flow	HUVEC	BAEC	In vivo
Morphology	Small cell area Random orientation Cobblestone morphology	Moderate cell area Random orientation Cobblestone morphology	Large cell area Aligned to flow Spindle-like morphology [14, 16, 17]	Large cell area Aligned to flow Spindle-like morphology [18, 20, 64, 67]	Moderate cell area Aligned to flow Spindle-like morphology [12, 13, 21, 22]
Motility	Small displacement	Small displacement	Large displacement	N/A	N/A
Proliferation rate	High	Moderate	Moderate	N/A	Low-moderate [4, 66]
Apoptosis rate	High	Low	Moderate	N/A	N/A
Protein and gene expression	No change	No change in transporters ZO-1↓ (WB, 4 dyne cm^−2^)	OCLN (no change)	ZO-1 (no change) [14] OCLN ↓ (WB, 10 & 20 dyne cm^−2^) [77]	N/A

Morphological analysis is quantified as cell area, inverse aspect ratio, and orientation angle with respect to the flow direction. Cell area: small (<700 µm^2^), moderate (700–1200 µm^2^), large (>1200 µm^2^). Orientation: random/cobblestone (IAR ~ 0.6, orientation ~ 45°), aligned to flow/spindle-like (IAR < 0.4, orientation < 20°). Displacement is defined as the distance between the current location and its original position: small (<50 µm), large (>50 µm). Proliferation rate is defined as the percent of all cells that divide per hour: low (<0.1% h^−1^), moderate (0.1–0.3% h^−1^), high (>0.3% h^−1^). Apoptosis rate is defined as the percent of all cells that divide per hour: low (<0.05% h^−1^), moderate (0.05–0.1% h^−1^), high (>1% h^−1^)

**Fig. 3:**
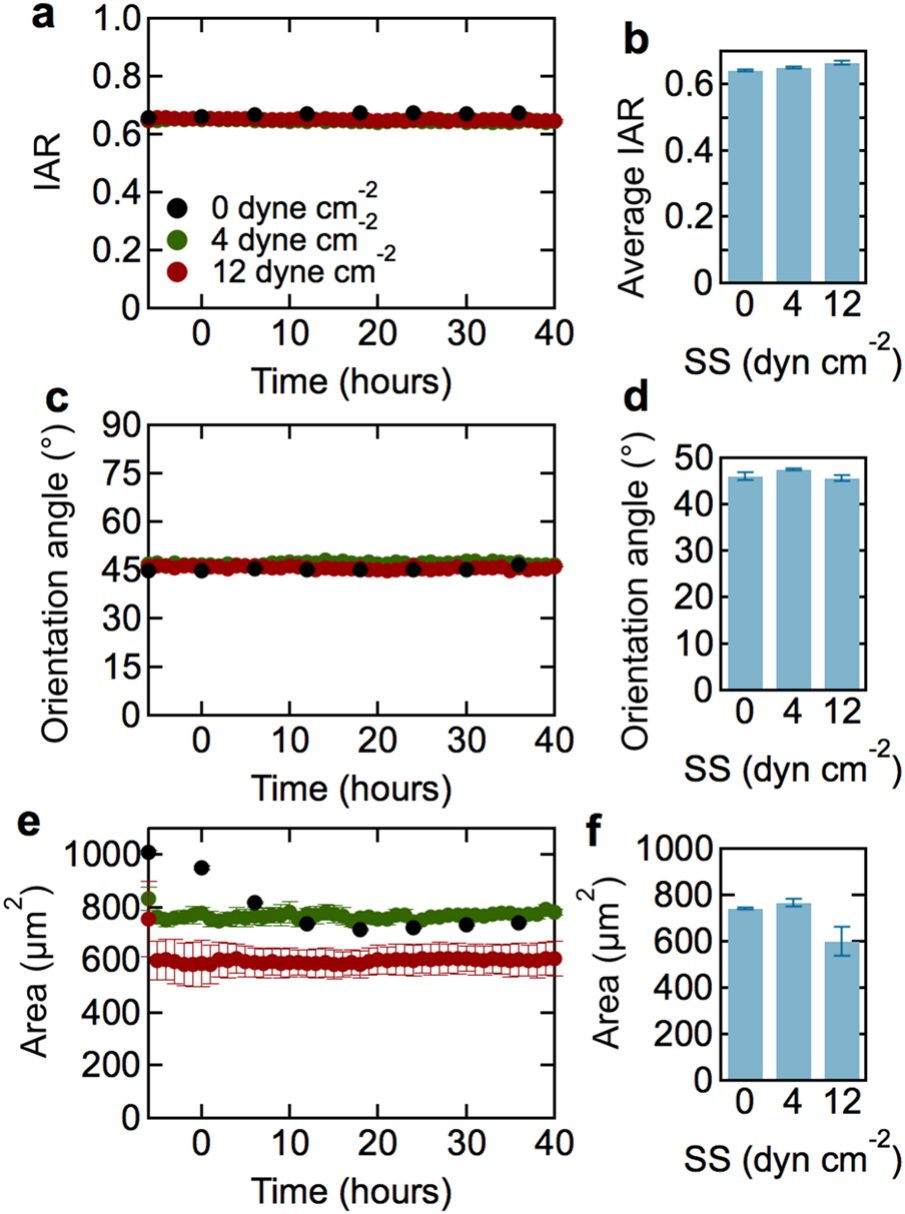
Morphological characterization of dhBMECs in confluent monolayers at 0 (static), 4, and 12 dyne cm^−2^ shear stress. **a** Average inverse aspect ratio (IAR) as a function of time. **b** Steady state IAR. **c** Average orientation angle as a function of time. **d** Steady state orientation angle. **e** Average cell area as a function of time. **f** Steady state cell area. Each data point represents approximately 18,000 cells over three independent experiments. Steady state values were obtained from the average values between 30 and 40 h. *Error bars* represent mean ± SE

To quantitatively characterize cell morphology, we measured the inverse aspect ratio (IAR), orientation angle, and cell area as a function of shear stress and time (Fig. 3). The IAR for dhBMEC monolayers under static conditions was about 0.65 and did not change with time (Fig. 3a). Under static and flow conditions, the average orientation angle of the dhBMEC monolayers remained close to 45°, corresponding to a random orientation of cells and showing that there was no cell alignment in response to shear stress (Fig. 3b). These results show that the dhBMECs do not elongate in response to physiological shear stress.

Changes in cell area reflect gross changes in cell turnover. Histograms of cell area (Additional file 4: Figure S2) show a log-normal distribution with a well-defined peak and a small number of cells that are considerably larger. Under 4 dyne cm^−2^, the average cell area was about 800 µm^2^ and remained approximately constant throughout the experiment (Fig. 3c). At 12 dyne cm^−2^, the average cell area was about 750 µm^2^ and also remained constant throughout the experiment. Under static conditions, the cell area decreased to a steady state value of about 750 µm^2^ after about 5 h. Despite these differences, there is no statistically significant difference in average area at 40 h between 0, 4, and 12 dyne^−2^ across all experiments analyzed.

Morphological changes to endothelial cells in response to shear flow are usually observed within 12–24 h [10, 13, 14, 16, 23, 24], therefore the experiments reported here were performed for 40 h. To verify the lack of a morphological response of dhBMECs at longer times, selected experiments were performed for 60 h under shear stress confirming that there is no further change in cell morphology (Additional file 4: Figure S3, Table S2).

In these experiments, cells were seeded at a density of 500,000 cells per channel. To ensure that seeding density did not influence steady state morphology, we also performed experiments at seeding densities of 250,000 and 125,000 cells per channel. Seeding at 250,000 cells per channel resulted in a longer time reach confluence, however, there was no difference in cell morphology (Additional file 4: Figure S4, Table S3). Seeding at 125,000 cells per channel did not result in the formation of a confluent monolayer.

### Rates of proliferation and apoptosis

**Fig. 4:**
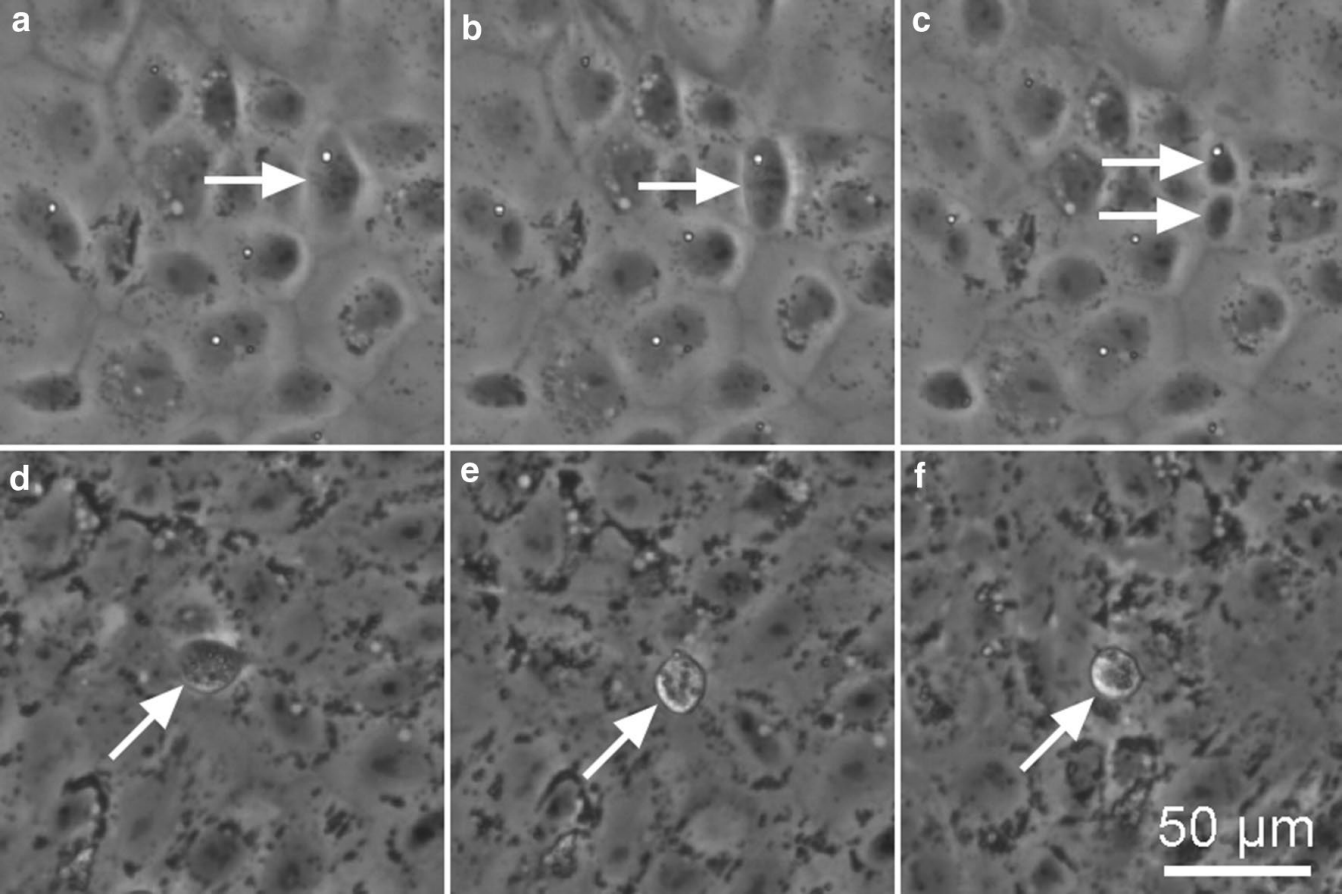
Representative phase contrast images of cell division and apoptosis events in confluent dhBMEC monolayers. **a**–**c** dhBMEC undergoing division over the course of 1 h. **d**–**f** dhBMEC undergoing apoptosis over an hour. Images were captured at 20 min intervals

**Fig. 5:**
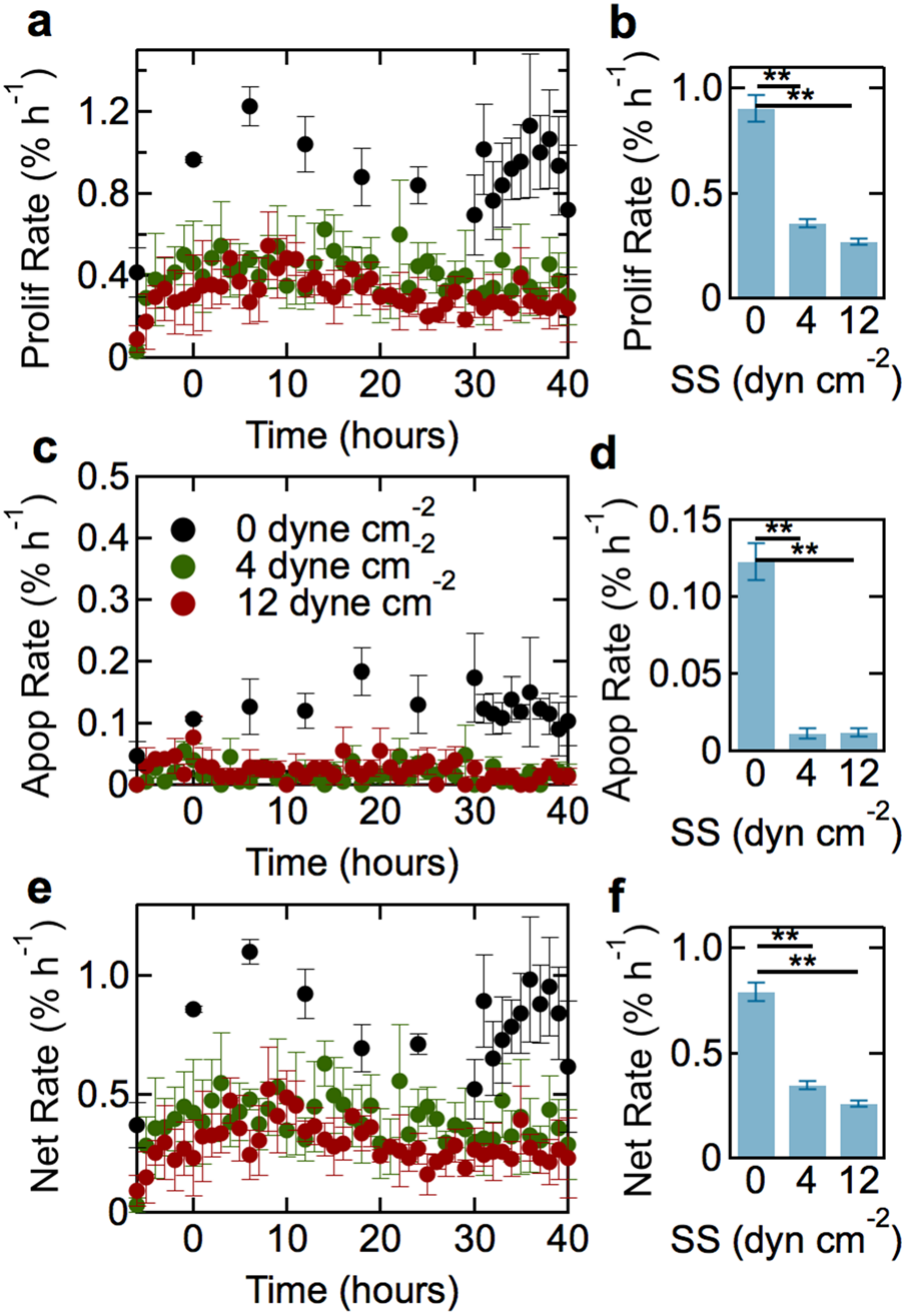
Proliferation and apoptosis rates for dhBMECs in confluent monolayers at 0 (static), 4, and 12 dyne cm^−2^. **a** Proliferation rate versus time. **b** Steady state proliferation rate as a function of shear stress. **c** Apoptosis rate as a function of time. **d**. Steady state apoptosis rate as a function of shear stress. **e** Net rate of change as a function of time. **f** Steady state net rate of change as a function of shear stress. Data obtained from analysis of approximately 18,000 cells over three independent experiments. Steady state values were obtained from the average rates between 30 and 40 h. *Error bars* represent mean ± SE

To assess the effect of shear stress on turnover, we visually detected proliferation and apoptosis events in phase contrast, time-lapse videos (Fig. 4). Relative turnover rates are usually measured using labeling probes (e.g. thymidine, EdU) that incorporate into the cell nucleus upon cell division [39–42]. Direct visualization provides direct, quantitative measurement of both proliferation and apoptosis rates, and enables monitoring in real time. The proliferation and apoptosis rates are reported as a percentage of the total number of cells per hour (Fig. 5). Under static conditions, the proliferation rate is around 1.0% h^−1^ (Fig. 5a, b). Under 4 dyne cm^−2^, the proliferation rate reaches a maximum of about 0.4% h^−1^ during the conditioning phase and gradually decreases to a steady state value of 0.35 ± 0.02% h^−1^. Similar results are obtained at 12 dyne cm^−2^, although the steady state value is somewhat smaller (0.27 ± 0.01% h^−1^).

The apoptosis rate under static conditions has a steady state value of 0.12% h^−1^ (Fig. 5c, d). Under shear stress at both 4 and 12 dyne cm^−2^, the apoptosis rate remained constant throughout the experiment with a steady state value of 0.01% h^−1^, an order of magnitude lower than under static conditions (Fig. 5c, d). The net rate of change in the number of cells within a monolayer, defined as the difference between the proliferation and apoptosis rates (Fig. 5e, f), is dominated by the larger proliferation rate.

**Fig. 6:**
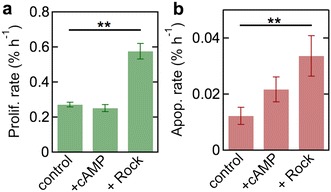
Steady state proliferation and apoptosis rates. **a** Steady state proliferation rate as a function of media condition at 12 dyne cm^−2^. **b** Steady state apoptosis rate as a function of media condition at 12 dyne cm^−2^. Steady state values are the average values obtained between 30 and 40 h. *Error bars* represent mean ± SE

To determine the effects of vascular modulators on steady state proliferation and apoptosis rates, we performed additional experiments at 12 dyne cm^−2^ where the endothelial cell media was supplemented with DB-cAMP or ROCK inhibitor (Fig. 6a, b). Cyclic-AMP (DB-cAMP) is an intracellular secondary messenger that has a variety of functions, and has been shown to increase barrier function and decrease proliferation and apoptosis rates in endothelial cells [42]. The addition of DB-cAMP had no effect on the steady state rates of proliferation and apoptosis, suggesting that the dhBMEC monolayers are already in a relatively quiescent state. The ROCK pathway mainly regulates cell shape and motility by acting on the cytoskeleton [43], but is commonly used to promote survival of iPSCs [44]. The addition of ROCK inhibitor significantly increased the proliferation rate from 0.27 to 0.57% h^−1^ and increased the apoptosis rate from 0.012 to 0.033% h^−1^. The increase in proliferation rate is larger than the increase in apoptosis rate, resulting in an increase in the net change in cell number on exposure to ROCK inhibitor from 0.26 to 0.54% h^−1^, consistent with increased survival.

### Cell motility

**Fig. 7:**
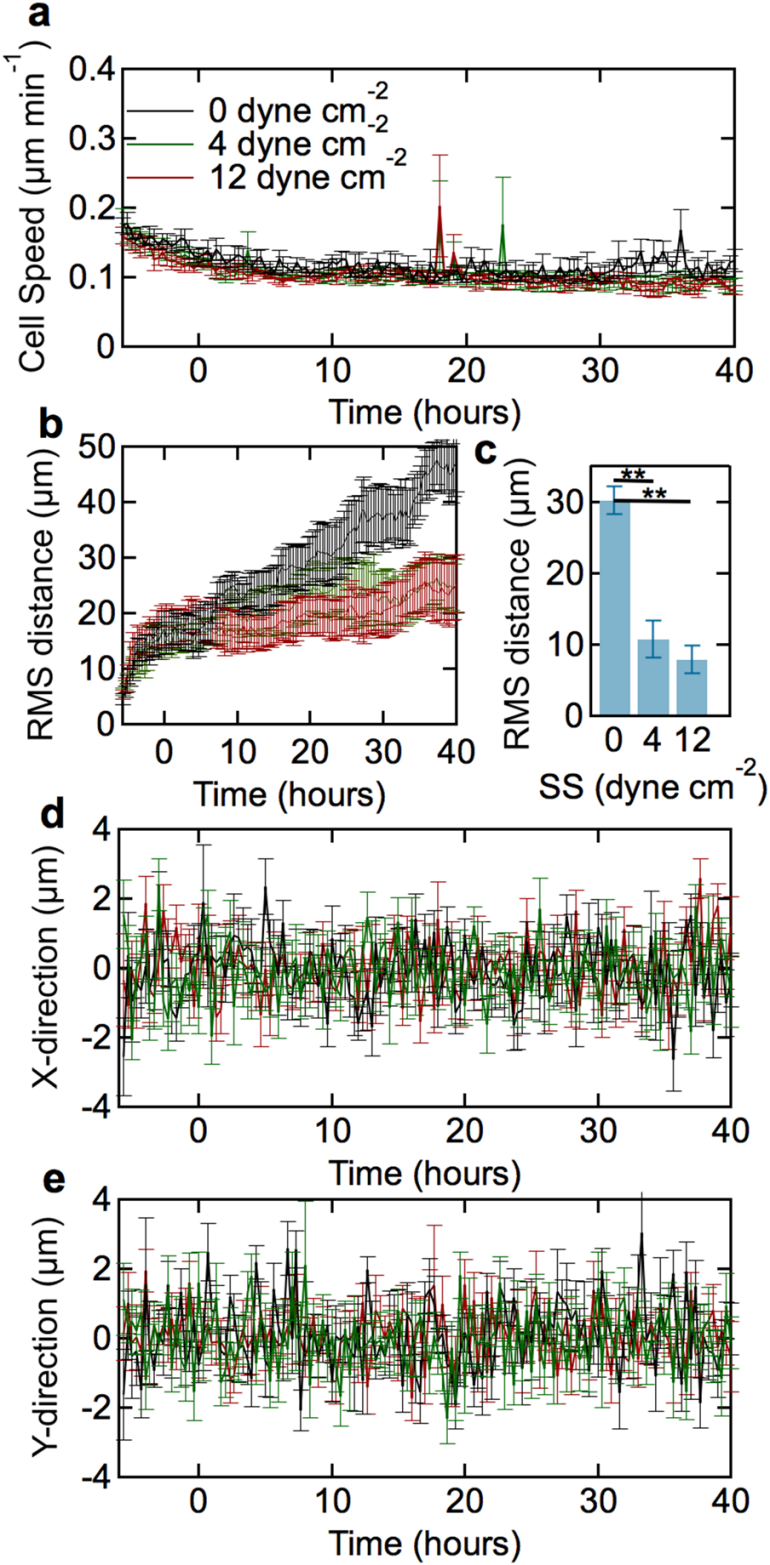
Average speed and displacement of dhBMECs in confluent monolayers at 0 (static), 4, and 12 dyne cm^−2^. **a** PIV cell speed as a function of time. Each point represents the average of 18,000 cells over three independent experiments. **b** RMS displacement as a function of time. **c** Total cell displacement between 0 and 40 h. **d** Directionality along the *x*-axis as a function of time. Positive *x*-axis corresponds to the direction of flow. **e** Directionality along the *y*-axis (perpendicular to the flow direction) as a function of time. Data for displacement and directionality were obtained from analysis of at least 100 cells over three independent experiments. *Error bars* represent mean ± SE

To assess cell motility, we measured the average cell speed, the RMS displacement, and the directionality. The average cell speed, a measure of cell activity [11, 26, 33], was calculated by automated particle image velocimetry (PIV) analysis [19]. The average speed within the monolayers decreased from a maximum of approximately 0.2 µm min^−1^ during the 6-h conditioning period, to a steady state value of about 0.1 µm min^−1^ under static conditions and under 4 and 12 dyne cm^−2^ shear stress (Fig. 7a).

The RMS displacement is a measure of translation within the monolayer and is calculated as the distance of the center of mass of the cell nucleus from an initial reference point. Under static conditions, the displacement increases monotonically with a slope of about 0.01 µm min^−1^, corresponding to 30 µm over the course of the experiment (Fig. 7b). Under shear stress, the displacement was about 15 µm during the initial 6-h conditioning period, but then increased very slowly during experiment (Fig. 7b). At both 4 and 12 dyne cm^−2^, the displacement under shear stress was about 10 µm over 40 h (Fig. 7c). We confirmed that there is no influence of flow on displacement within the monolayer by measuring the x- and y- components of the directionality (Fig. 7d, e).

### Expression of BBB markers

To assess changes in protein and gene expression of dhBMECs in confluent monolayers in response to shear stress, immunofluorescence staining, western blot and qPCR were performed after 40 h under static conditions (0 dyne cm^−2^) and at 4 and 12 dyne cm^−2^.

### Immunofluorescence imaging

**Fig. 8:**
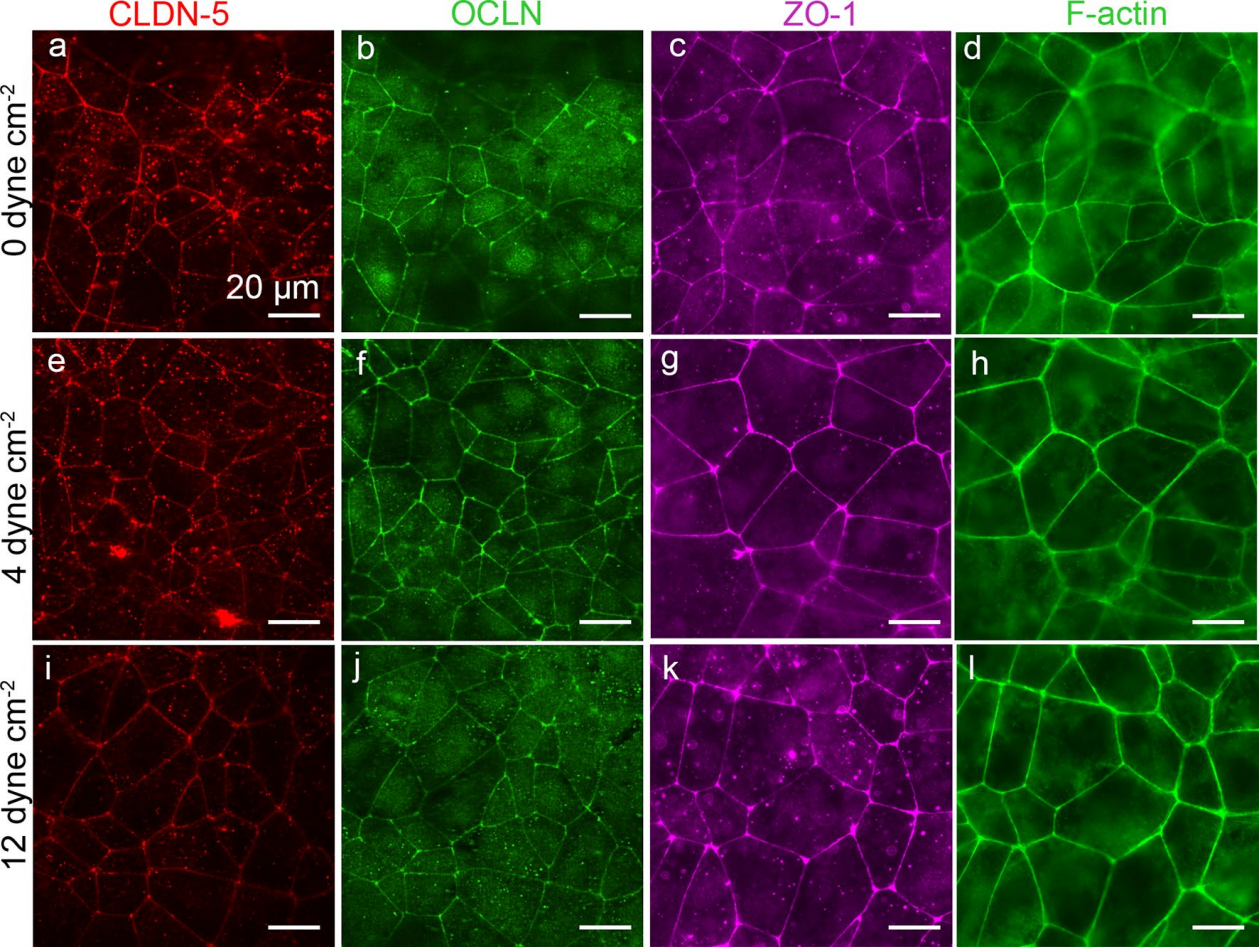
Representative immunofluorescence images of dhBMEC monolayers fixed and stained after 40 h at 0, 4, and 12 dyne cm^−2^. **a**, **e**, **i** CLDN-5. **b**, **f**, **j** OCLN. **c**, **g**, **k** ZO-1. **d**, **h**, **l** F-actin. Note that CLDN-5/OCLN and ZO-1/f-actin were obtained for different monolayers

To evaluate the expression and localization of tight junction and cytoskeletal proteins, monolayers were stained for claudin-5, occludin, zonula occludens 1 (ZO-1), and F-actin (Fig. 8). Claudin-5 and occludin are transmembrane tight junction proteins that bind to the PDZ domain and associate with the actin cytoskeleton [45]. ZO-1 is a peripheral junctional protein that is part of the PDZ domain and links occludin directly to the cortical actin skeleton [45, 46]. Under static conditions, claudin-5, occludin, and ZO-1 are localized to cell–cell junctions (Fig. 8a–c). The cell boundaries are generally straight resulting in a well-defined polygonal network, consistent with previous reports of dhBMEC monolayers [4–6, 47–49]. In contrast, tight junction stains for immortalized and primary BMECs from humans and animals often show elongated cells with junctions that are often serrated [50–52]. There are no clear differences between claudin-5, occludin, and ZO-1 stains under static and flow conditions, suggesting that tight junction networks are already well established under static conditions. The junctional network also shows that there is no elongation and alignment under flow, as described previously.

F-actin is a cytoskeletal protein that plays an important role in cell motility, cell shape, and the maintenance of cell junctions [53]. After 40 h at 0, 4, or 12 dyne cm^−2^, F-actin is highly localized to the peripheral regions of the cell, near the cell–cell junctions and few stress fibers were seen within the cell (Fig. 8d, h, l). F-actin remained randomly oriented in all conditions and did not align parallel to flow. Quantitative analysis of the intensity of claudin-5, occludin, ZO-1, and F-actin expression at the cell–cell junctions revealed no significant differences between static and flow conditions (Additional file 4: Figure S5). The endothelial cell nuclei maintain an oval shape under all conditions (Additional file 4: Figure S6).

### Western blot

**Fig. 9:**
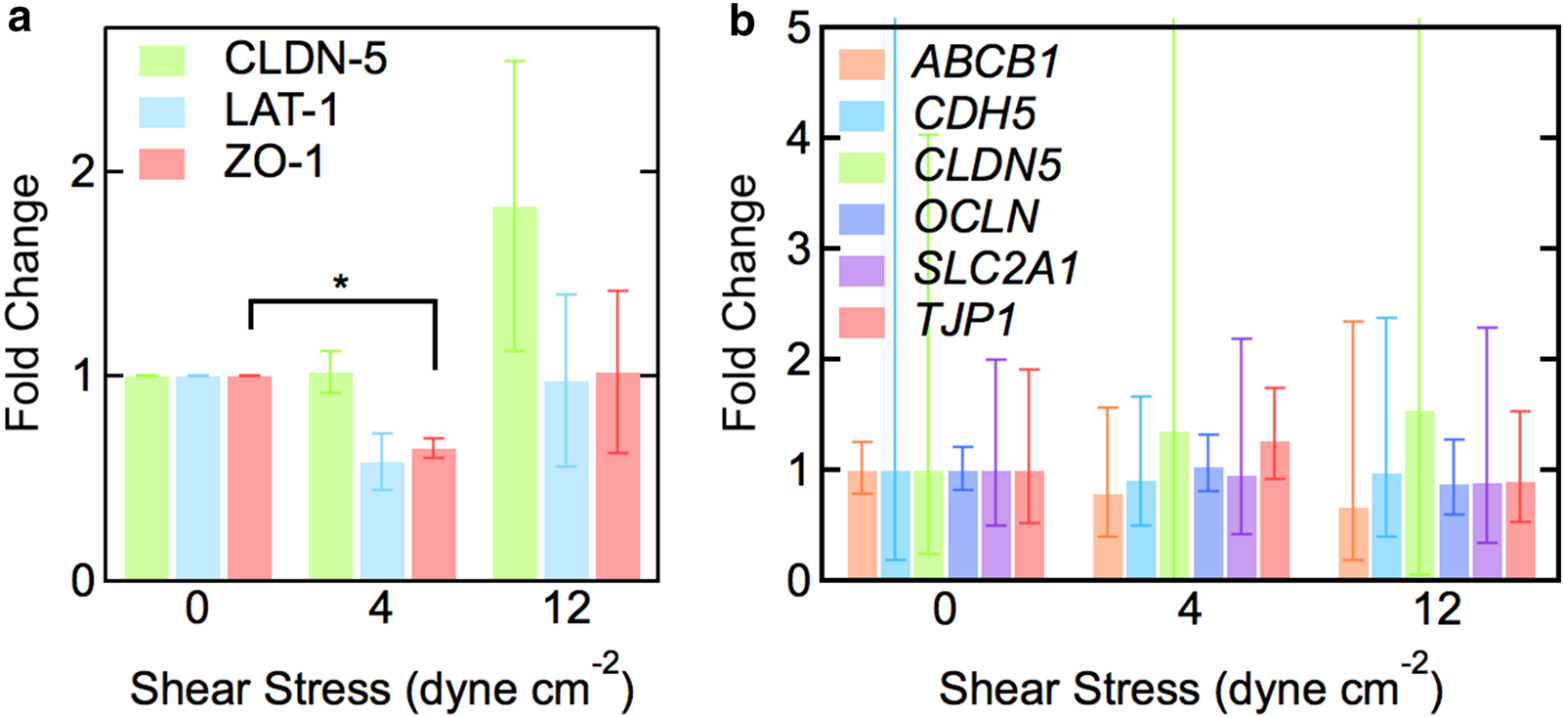
Protein and gene expression of dhBMECs in confluent monolayers after 40 h at 0, 4, and 12 dyne cm^−2^shear stress. **a** Relative intensities of protein expression of CLDN-5, LAT-1, and ZO-1 using western blot analysis. Data were obtained from analysis of four different differentiations for CLDN-5 and LAT-1 and three differentiations for ZO-1. Fold changes are reported with respect to static conditions (0 dyne cm^−2^). β-actin was used as a control. Error bars represent SE. *Asterisk* represents p < 0.05. **b** Relative gene expression of ABCB1 (P-gp), CDH5 (VE-cad), CLDN5 (claudin-5), OCLN (occludin), SLC2A1 (GLUT-1), and TJP1 (ZO-1) from qPCR. Data were obtained from analysis of three separate differentiations. Fold changes are reported with respect to static conditions (0 dyne cm^−2^). *Error bars* represent mean ± SE. ACTB and GAPDH were used as the housekeeping genes

To determine whether protein level expression of key BBB proteins changes in response to shear stress, western blots were performed for claudin-5 (CLDN-5), large amino acid transporter 1 (LAT-1), and ZO-1 after 40 h at 0, 4, or 12 dyne cm^−2^ (Fig. 9a; Additional file 4: Figure S7). Claudin-5 is a tight junction protein that is highly expressed in the brain and responsible for maintaining proper blood–brain barrier function [54]. LAT-1 is a large neutral amino acid transporter that is highly expressed in the brain [55]. There were no significant differences in CLDN-5 or LAT-1 expression levels under shear stress compared to static conditions, and no difference between low and high shear stress. Although the mean expression of claudin-5 increased almost twofold at 4 dyne cm^−2^ compared to static conditions, the difference is not statistically significant (p > 0.05). The level of LAT-1 expression at 4 dyne cm^−2^ is lower than under static conditions but also not statistically significant (p > 0.05). ZO-1 expression at 4 dyne cm^−2^ is statistically lower than static conditions (0 dyne cm^−2^), but there is no statistical difference between ZO-1 expression at 4 and 12 dyne cm^−2^.

### Gene expression

To examine the impact of shear stress on gene expression of important blood–brain barrier proteins, we determined the relative expression of several transporters (*ABCB1*, *SLC2A1*) and tight junction and junctional proteins (*CDH5*, *CLDN5*, *OCLN*, *TJP1*) (Fig. 9b). *ABCB1* (P-gp) is the gene for the P-glycoprotein efflux pump [56]. *SLC2A1* is the gene for the GLUT-1 transporter that transports glucose across the blood–brain barrier, and is highly expressed in brain capillary endothelium [57]. *CDH5* (VE-cad) is the gene for vascular endothelial cadherin (VE-cadherin), an endothelial-specific cadherin and adherens junction protein that links adjacent cells together and plays an important role in vascular homeostasis [58]. *CLDN5* encodes for the tight junction protein claudin-5 that is highly expressed in BMECs [54]. *OCLN* encodes occludin, a membrane-spanning tight junction protein that connects adjacent cells to each other and is highly expressed in the brain [59]. *TJP1* is the gene for ZO-1, a tight junction protein that is localized to tight junctions and links the transmembrane tight junction protein occludin to the cytoskeleton [60].

There were no significant differences in gene expression of transporters (*ABCB1*, *SLC2A1*) or junctional proteins (*CDH5*, *CLDN5*, *OCLN*, *TJP1*) at 4 and 12 dyne cm^−2^ compared to static conditions (0 dyne cm^−2^). *CDH5* and *CLDN5* exhibit high standard error in fold change due to batch-to-batch variability between different differentiations (Additional file 4: Figure S8). These differences may originate from differential expression of these proteins due to variations in tight junction formation between differentiations (Additional file 4: Figure S8). Changes in gene expression of *CDH5* and *CLDN5* due to shear stress within individual differentiations also revealed no trend.

## Discussion

### Cell morphology

Elongation and alignment in response to shear stress is a hallmark of endothelial cells in large vessels [8, 14, 16, 23, 24, 61–63]. In 2D cell culture, confluent monolayers of human umbilical vein endothelial cells (HUVECs), bovine aortic endothelial cells (BAECs), porcine pulmonary artery ECs, and primary baboon arterial endothelial cells (pBAECs) under physiological shear stress undergo a transition from a cobblestone morphology to an elongated spindle-like morphology and align in the direction of flow, recapitulating EC morphology in larger vessels [7, 8, 10, 11, 14–19]. In previous work, we have shown that immortalized hBMECs do not elongate or align in response to physiological shear stress [19]. Here we show that, similarly, iPSC-derived hBMECs do not elongate and align in response to shear stress, providing further evidence that this is a unique phenotype of brain microvascular endothelial cells. The average cell area for dhBMECs is considerably smaller than for HUVECs, which is in the range 1500–2000 µm^2^ [19], and around 1200 µm^2^ for BAECs [64]. In previous work we have shown that the area for immortalized hBMECs is 800–1500 µm^2^ and increases with increasing shear stress [19].

### Proliferation and apoptosis

The rates of proliferation and apoptosis for dhBMECs decrease significantly under shear stress. The proliferation rate decreases by about threefold and the apoptosis rate by more than tenfold compared to static conditions. The net turnover rate (proliferation rate–apoptosis rate) under steady state conditions is 0.8% h^−1^ under static conditions, but decreases with increasing shear stress, to 0.3% h^−1^ at 4 dyne cm^−2^ and 0.2% h^−1^ at 12 dyne cm^−2^.

The net turnover rate reflects any significant changes in cell area and hence is a measure of stress on the monolayer. For example, large positive values can lead to the formation of mounds or overgrowth, while large negative values can lead to gaps in the monolayer. The positive net turnover rate corresponds to an increase in the number of cells over time, however, this increase is not sufficiently large to cause a measurable change in the average cell area. Under steady state conditions (30–40 h) we can expect the monolayer to increase the number of cells by 8, 3, and 2% at 0, 4, and 12 dyne cm^−2^, respectively. Therefore, the expected decrease in average cell area is within the variation and is not detected.

The net turnover rate of 0.2–0.3% h^−1^ under shear stress is similar to values for HUVEC monolayers (0.1% h^−1^), and 3D microvessels (0.25–0.6% h^−1^; labeling index) [65]. Surprisingly little is known about the turnover of hBMECs in vivo, however, results from thymidine labeling in mice suggest rates of about 0.04% h^−1^, about an order of magnitude lower than endothelial cells in other tissues [39–41, 66, 67].

### Cell motility and displacement

The average speed of dhBMECs under shear stress is around 0.1 µm min^−1^, lower than values for both HUVECs and immortalized hBMECs, typically around is 0.2 µm min^−1^ [19]. More importantly, the average cell displacement in dhBMEC monolayers is extremely low, around 15 µm over 40 h. In contrast, HUVECs under the same conditions show a displacement of 200–500 µm over 40 h under shear stress, an increase of more than 100-fold compared to the dhBMECs. The very small displacement observed for dhBMECs could arise from increased adhesion to the substrate or increased cell–cell adhesion. Since dhBMEC monolayers are relatively easy to displace from the substrate as sheets of cells, the low displacement is likely due to increased cell–cell adhesion. As described previously, dhBMECs in confluent monolayers cells appear to flatten under shear stress. There is no change in cell area and hence if the cell volume remains constant, then the flattening must be a result of increased overlap between cells. Increased cell–cell overlap would increase the strength of cell–cell junctions and explain the very low cell displacement. Ultrastructural studies of capillaries in animal models show substantial cell–cell overlap at tight junctions which may be important for maintaining low blood–brain barrier permeability [68, 69]. These results suggest that an important role of flow may be in increasing the contact area between cells which in turn enhances barrier function.

### Protein and gene expression

Immunofluorescence images revealed no difference in the expression and localization of claudin-5, occludin, ZO-1, or F-actin in response to flow, suggesting that tight junctions are established under static conditions [70]. In contrast, bovine brain microvascular endothelial cells under 10 dyne cm^−2^ shear stress for 24 h showed increased localization of tight junction proteins to the cell–cell borders [71].

The ability of cells to sense and adapt to their environment is crucial, and the mechanosensing responses to shear stress and other mechanical forces are mediated by the actin cytoskeleton [72]. In dhBMEC monolayers, F-actin is localized to the cell–cell junctions and we do not see any significant stress fibers within the cell body. In contrast, other ECs such as HUVECs and BAECs, show significant cytoskeleton reorganization with alignment of stress fibers parallel to the direction of flow [73–75]. Stress fibers formed in vivo in cardiac vascular endothelial cells are also aligned parallel to the direction of flow and are thought to be necessary to withstand high hemodynamic stresses [76]. These results suggest that elongation and alignment is coupled with cytoskeleton reorganization, neither of which are observed in dhBMEC monolayers.

Shear stress did not induce any changes in expression of several BBB markers at the protein or gene level. The fact that there were no changes in expression of BBB markers with shear stress is coupled with the fact that there is no morphological transition (cobblestone to spindle-like). Previous in vitro studies with bovine and human brain microvascular endothelial cells have shown up-regulation of various junctional and transporter genes in response to shear stress [77, 78]. In contrast to other cell lines, dhBMECs under static conditions exhibit transendothelial electrical resistance values in excess of 2000 Ω cm^2^ [5, 6, 79], comparable to values reported in vivo in rat brains (1000–1500 Ω cm^2^) [80]. These results suggest that the tight junction architecture in dhBMECs is already established during monolayer formation under static conditions, and that flow is not necessary for this process. This conclusion is supported by the fact that very high TEER vales are obtained for confluent monolayers on transwell supports under static conditions [5, 6, 79]. As described previously, we hypothesize that flow serves to increase the contact area between cells, resulting in very low cell displacement and preventing the morphological transition that is thought to be a hallmark of ECs.

## Conclusions

Shear stress plays an important role in modulating endothelial cell morphology, structure and function. Here we show that dhBMECs exhibit a unique phenotype in response to shear stress: (1) they do not elongate and align, (2) the displacement of individual cells within the monolayer over time is significantly decreased, (3) the rates of proliferation and apoptosis decrease, (4) there is no cytoskeletal reorganization or formation of stress fibers within the cell, and (5) there is no change in expression levels of key blood–brain barrier markers. This response is very different to the response of endothelial cells from other tissues, indicating that the dhBMEC have a unique phenotype in response to shear stress that may be important in maintenance of the blood–brain barrier. Since the blood–brain barrier has specialized endothelial cells with tight junctions that minimize paracellular transport and specialized transporters to regulate transport across the brain, our results suggest that these endothelial cells may also have a unique response to shear stress. The implications of this work are that: (1) in confluent monolayers of dhBMECs, tight junctions are well formed under static conditions, (2) the formation of tight junctions decreases cell motility, compared to other ECs, and hence prevents any morphological transitions, (3) flow serves to increase the contact area between cells, resulting in very low cell displacement in the monolayer, (4) since tight junctions are already formed under static conditions, increasing the contact area between cells does not cause upregulation in protein and gene expression of BBB markers, and (5) the increase in contact area induced by flow makes barrier function more robust. These unique features of dHBMECs as compared to other endothelial cell lines may contribute to the unique tightness and highly selective permeability of the blood–brain barrier. Shear stress is one of many parameters that influence endothelial phenotype. Therefore, this work contributes to the emerging understanding of factors that are important in developing accurate in vitro models of the blood–brain barrier.

## Additional files

**Additional file 1.** dhBMEC monolayer under 4 dyne cm^−2^.

**Additional file 2.** dhBMEC monolayer under 12 dyne cm^−2^.

**Additional file 3.** dhBMEC monolayer under 0 dyne cm^−2^.

**Additional file 4: Supplementary Information.** Figure S1. PIV cell speed validation. **Table S1.** Primary antibodies used for staining for immunofluorescence (IF) and western blot (WB). **Figure S2.** Probability density cell area histogram. **Figure S3.** Steady state morphology of dhBMEC monolayers over 60 h. **Table S2.** Steady state morphology of dhBMEC monolayers over 40 hours compared to 60 hours. **Figure S4.** Steady state morphology of confluent monolayers of dhBMECs under shear stress seeded at 250,000 cells per channel. **Table S3.** Role of seeding density on cell morphology in confluent monolayers of dhBMECs under shear stress. **Figure S5.** Quantification of selected markers at cell-cell junctions. **Figure S6.** Morphology of dhBMEC nuclei. **Figure S7.** Western blots. **Figure S8.** Gene expression variability in the dhBMEC differentiation protocol and qPCR preparation process.

## Abbreviations

ABCB1: ATP binding cassette subfamily B member 1
ACTB: beta actin
BAEC: bovine aortic endothelial cell
BBB: blood–brain barrier
BBMvEC: bovine brain microvascular endothelial cell
BC1-hBMEC: BC1-derived human brain microvascular endothelial cell
bFGF: basic fibroblast growth factor
BMEC: brain microvascular endothelial cell
CDH5: cadherin 5/VE-cadherin
CLDN5: claudin-5
DAPI: 4′,6-diamidino-2-phenylindole fluorescent stain
EC: endothelial cell
GAPDH: glyceraldehyde 3-phosphate dehydrogenase
GLUT1: glucose transporter 1
hBMEC: human brain microvascular endothelial cell
hiPSC: human induced pluripotent stem cell
HUVEC: human umbilical vein endothelial cell
IAR: inverse aspect ratio
ID: inner diameter
OCLN: occludin
pBAEC: primary baboon artery endothelial cell
PBS: phosphate buffered saline
PDMS: polydimethylsiloxane
P-gp: p-glycoprotein
PIV: particle image velocimetry
qPCR: quantitative polymerase chain reaction
RIPA: radio immunoprecipitation assay
ROCK: rho-associated protein kinase
SLC2A1: solute carrier family 2 member 1
TEER: transendothelial electrical resistance
TJP1: tight junction protein 1/gene for ZO-1
UM/F-: unconditioned media without bFGF
VECAD: VE-cadherin
ZO1: zonula occludens 1
