# Supplementary material for: Effect of shear stress on iPSC-derived human brain microvascular endothelial cells (dhBMECs)
Source: Fluids Barriers CNS. 2017 Aug 4;14:20. doi: 10.1186/s12987-017-0068-z (PMC5543552; doi:10.1186/s12987-017-0068-z)
Supplement: Supplementary file 4 — Additional file 4: Supplementary Information. Figure S1. PIV cell speed validation. Table S1. Primary antibodies used for staining for immunofluorescence (IF) and western blot (WB). Figure S2. Probability density cell area histogram. Figure S3. Steady state morphology of dhBMEC monolayers over 60 h. Table S2. Steady state morphology of dhBMEC monolayers over 40 hours compared to 60 hours. Figure S4. Steady state morphology of confluent monolayers of dhBMECs under shear stress seeded at 250,000 cells per channel. Table S3. Role of seeding density on cell morphology in confluent monolayers of dhBMECs under shear stress. Figure S5. Quantification of selected markers at cell-cell junctions. Figure S6. Morphology of dhBMEC nuclei. Figure S7. Western blots. Figure S8. Gene expression variability in the dhBMEC differentiation protocol and qPCR preparation process. [file 12987_2017_68_MOESM4_ESM.docx]

**Supporting Information**

# Effect of Shear Stress on iPSC-derived human brain microvascular endothelial cells (dhBMECs)

Jackson G. DeStefano,* Zinnia S. Xu,* Ashley J. Williams, Nahom Yimam, Peter C. Searson

**Figure S1.** PIV cell speed validation.

**Table S1.** Primary antibodies used for staining for immunofluorescence (IF) and western blot (WB).

**Figure S2.** Probability density cell area histogram.

**Figure S3.** Steady state morphology of dhBMEC monolayers over 60 h.

**Table S2.** Steady state morphology of dhBMEC monolayers over 40 hours compared to 60 hours.

**Figure S4.** Steady state morphology of confluent monolayers of dhBMECs under shear stress seeded at 250,000 cells per channel.

**Table S3.** Role of seeding density on cell morphology in confluent monolayers of dhBMECs under shear stress.

**Figure S5.** Quantification of selected markers at cell-cell junctions.

**Figure S6.** Morphology of dhBMEC nuclei**.**

**Figure S7.** Western blots.

**Figure S8.** Gene expression variability in the dhBMEC differentiation protocol and qPCR preparation process.

**Supplemental Video V1.** dhBMEC monolayer under 4 dyne cm^-2^.

**Supplemental Video V2.** dhBMEC monolayer under 12 dyne cm^-2^.

**Supplemental Video V3.** dhBMEC monolayer under 0 dyne cm^-2^.


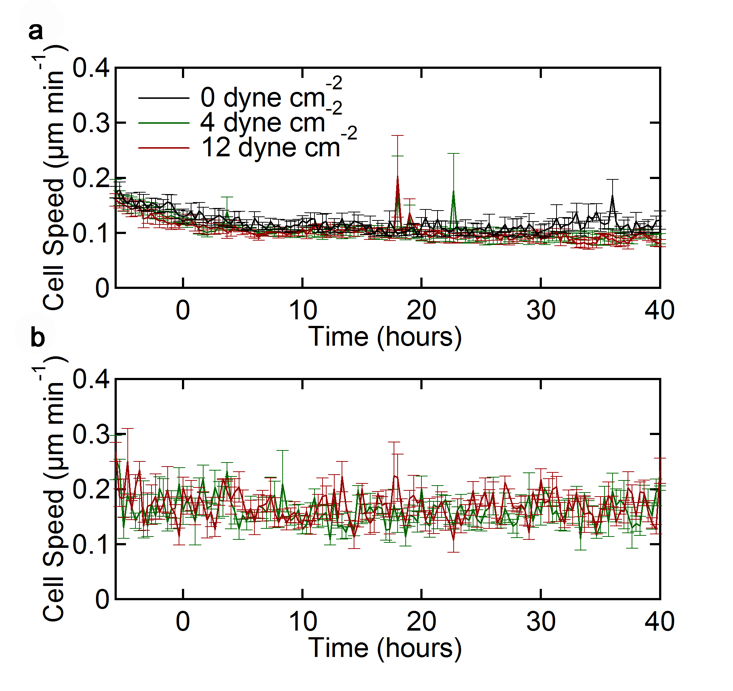


**Figure S1. PIV cell speed validation. a.** PIV cell speed as a function of time analyzed using OpenPIV (see *Methods* for details). **b.** Cell speed as a function of time analyzed manually from the position of the cell nuclei in each frame. N = 18,000 cells for automated analysis by OpenPIV, representing at three locations from three independent experiments. N = 90 cells for manual analysis, representing 10 cells traced in each of three frames over three independent experiments. There is excellent agreement between the two methods.

| Target Antigen | Antibody Description | Vendor | Product Number | Dilution |
| --- | --- | --- | --- | --- |
| ZO-1 | rabbit polyclonal | Thermo Fisher Scientific | 40-2200 | 1:100 IF  1:1500 WB |
| CLDN-5 | mouse monoclonal | Thermo Fisher Scientific | 35-2500 | 1:100 IF  1:1500 WB |
| OCLN | rabbit polyclonal | Thermo Fisher Scientific | 40-4700 | 1:100 IF |
| LAT-1 | rabbit polyclonal | Santa Cruz | sc-134994 | 1:1500 WB |
| β-actin | rabbit polyclonal | Cell Signaling Technologies | 4967S | 1:3000 WB |
| Goat Anti-Mouse IgG HRP |  | Bio-Rad | 1706516 | 1:1500 WB |
| Goat Anti-Rabbit IgG HRP |  | Bio-Rad | 1721019 | 1:1500 WB |

**Table S1. Primary antibodies used for staining for immunofluorescence (IF) and western blot (WB).**


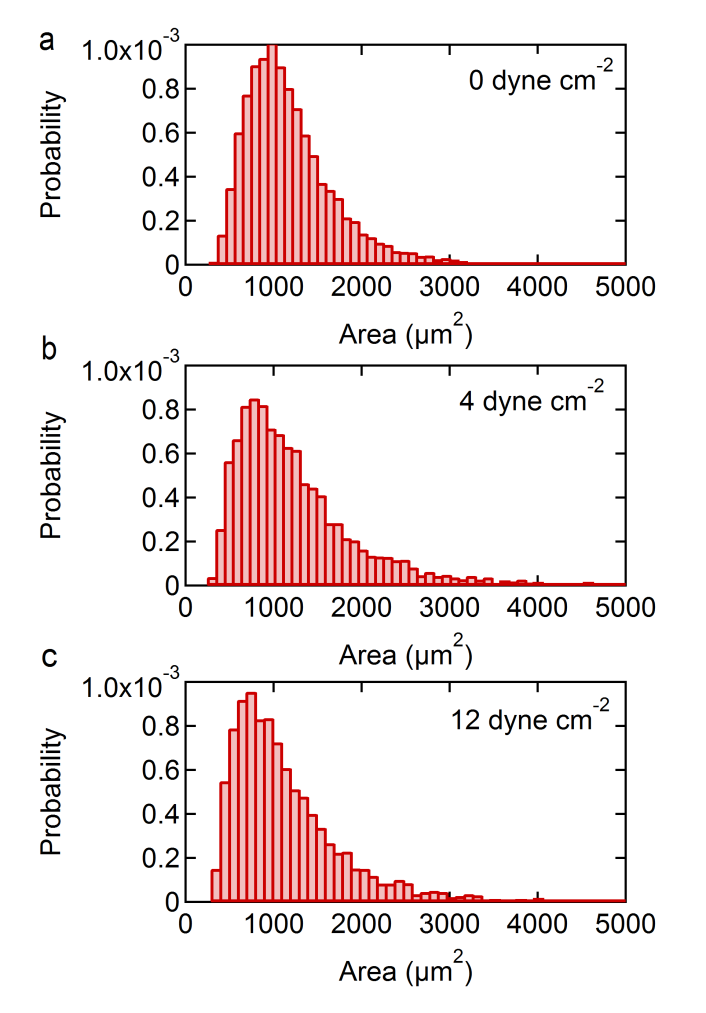


**Figure S2. Probability density cell area histogram**. Distribution of a cell area of confluent dHBMEC monolayers at 40 h under varying shear stress. Data shows a concentration of cells with area centered around 100 µm^2^ with larger cells less frequent. Distributions follow a log-normal distribution. **a.** 0 dyne cm^2^. **b.** 4 dyne cm^2^. **c.** 12 dyne cm^2^.


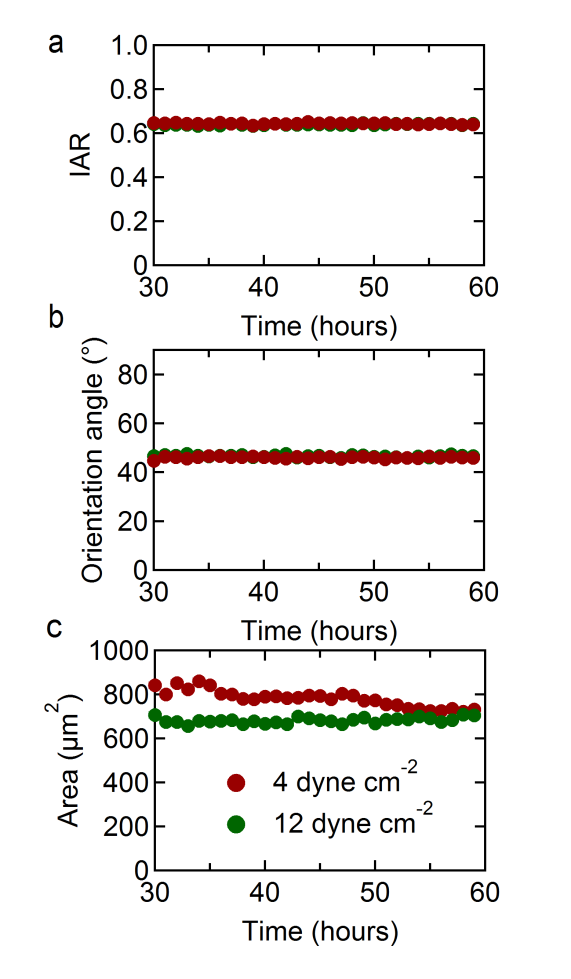


**Figure S3. Steady state morphology of dhBMEC monolayers over 60 h.** To ensure that steady state morphology was attained within 40 h, we performed an experiment on confluent monolayers for a duration of 60 h. Steady state morphological parameters were obtained from 30 – 60 h. **a**. Average IAR as a function of time. **b**. Average orientation angle as a function of time. **c**. Average area as a function of time. These results show that over 60 there is no change in cell morphology compared to experiments performed for 40 h (see Table below).

| **Parameter** (Mean ± SE) |  | **30 – 40 h** | **30 – 60 h** |
| --- | --- | --- | --- |
| **IAR** | 4 dyne cm^-2^ | 0.65 ± 0.002 | 0.64 ± 0.0006 |
|  | 12 dyne cm^-2^ | 0.66 ± 0.007 | 0.64 ± 0.0005 |
| **orientation angle (degrees)** | 4 dyne cm^-2^ | 47 ± 0.3 | 46 ± 0.08 |
|  | 12 dyne cm^-2^ | 45 ± 0.6 | 46 ± 0.08 |
| **cell area (µm^2^)** | 4 dyne cm^-2^ | 774 ± 2 | 781 ± 7 |
|  | 12 dyne cm^-2^ | 724 ± 1 | 682 ± 2 |

**Table S2. Steady state morphology of dhBMEC monolayers over 40 hours compared to 60 hours.** Values for 30 – 40 h represent average ± standard error over three imaging locations in three independent experiments. Values for 30 - 60 h represent average ± standard error over three imaging locations in one experiment.


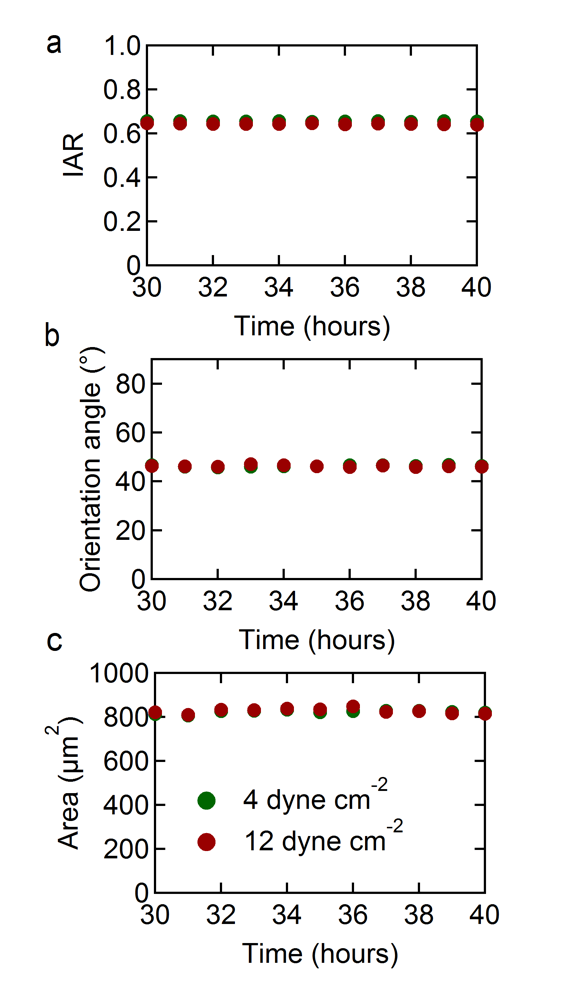


**Figure S4. Steady state morphology of confluent monolayers of dhBMECs under shear stress seeded at 250,000 cells per channel.** Cell inverse aspect ratio (IAR), orientation angle, and cell area for monolayers seeded at approximately 250,000 cells per channel. **a**. Average IAR as a function of time. **b**. Average orientation angle as a function of time. **c**. Average area as a function of time. To determine if seeding density had any impact on EC morphology we seeded cells at 125,000 cells per channel and 250,000 cells per channel. At 125,000 cells per channel, the dhBMECs did not form a confluent monolayer. A seeding density of 250,000 cells per channel is half the seeding density used for all other experiments reported here. These results show that the seeding density has no significant influence on steady state IAR or orientation angle in dhBMEC monolayers at 4 and 12 dyne cm^-2^ (see Table below).

**
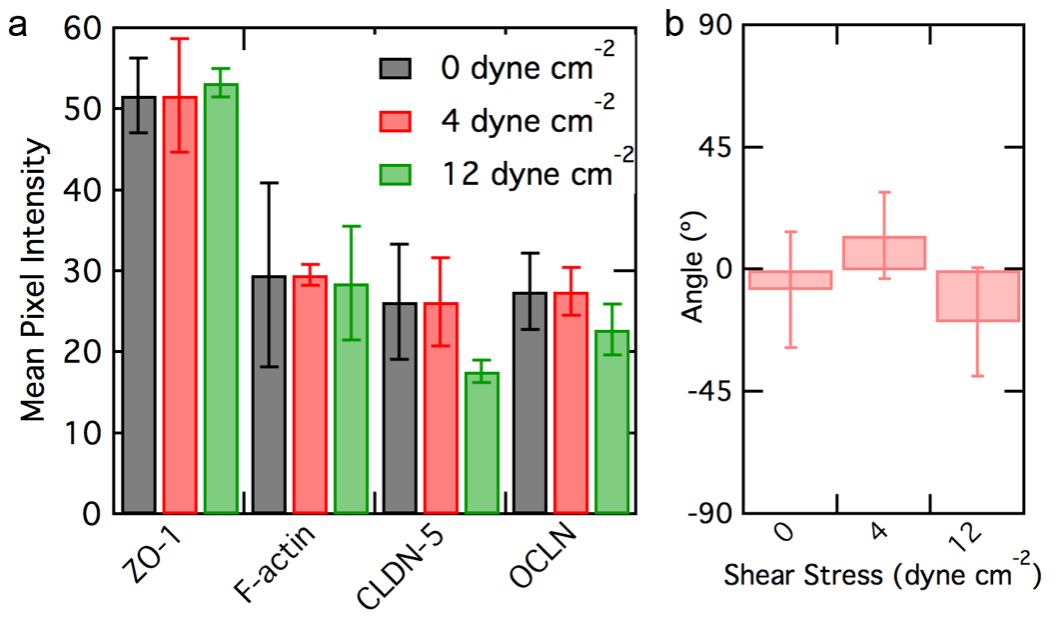
**

**Figure S5. Quantification of selected markers at cell-cell junctions.** Immunofluorescence images were quantified for relative intensity of selected markers at 0, 4, and 12 dyne cm^-2^. **a.** Relative intensities of CLDN-5, OCLN, ZO-1 and F-actin. Error bars represent mean ± SD. To assess junctional expression, cell-cell boundaries were traced using ImageJ (from one edge of the image field to the other edge three times per image) and average pixel intensity minus the background was collected and averaged. Mean pixel intensities did not vary significantly between the shear stress conditions for any of the junctional proteins or F-actin, suggesting that there was no change in localization or expression of junctional proteins with the addition of shear stress. **b.** F-actin fiber orientation. Error bars represent mean ± SE. F-actin fiber orientation was assessed using FibrilTool (Boudaoud et al., *Nat Protoc*. 2014). F-actin fibers were highly localized to the cell junctions, in contrast to HUVECs where stress fibers spanning the cell body and become aligned with the direction of flow.

**Table S3. Role of seeding density on cell morphology in confluent monolayers of dhBMECs under shear stress.** Values for 500,000 cells (normal seeding density) represent average ± standard error over three imaging locations in three independent experiments. Values for 250,000 cells (half seeding density) represent average ± standard error over three imaging locations in one experiment.

| **Parameter** (Mean ± SE) |  | **250,000 cells** | **500,000 cells** |
| --- | --- | --- | --- |
| **IAR** | 4 dyne cm^-2^ | 0.65 ± 0.002 | 0.65 ± 0.002 |
|  | 12 dyne cm^-2^ | 0.64 ± 0.002 | 0.66 ± 0.007 |
| **orientation angle (degrees)** | 4 dyne cm^-2^ | 46 ± 0.3 | 47 ± 0.3 |
|  | 12 dyne cm^-2^ | 46 ± 0.4 | 45 ±0.6 |
| **cell area (µm^2^)** | 4 dyne cm^-2^ | 823 ± 2 | 774± 2 |
|  | 12 dyne cm^-2^ | 827 ± 4 | 724 ± 1 |

**
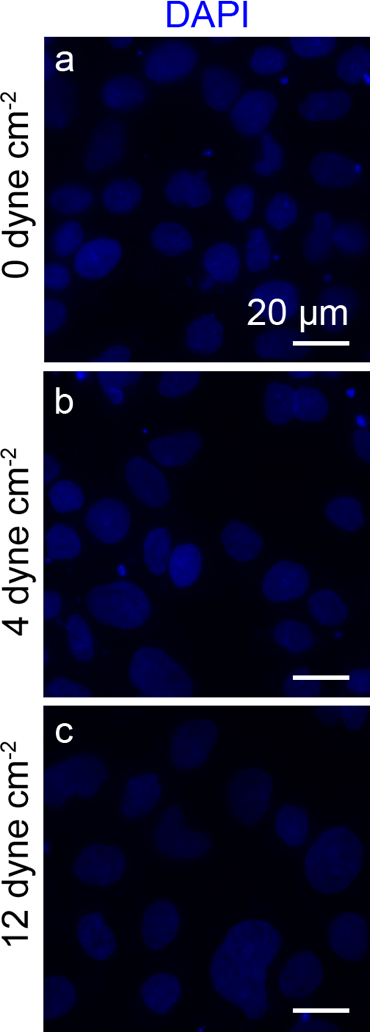
**

**Figure S6. Morphology of dhBMEC nuclei.** DAPI nuclear stains of dhBMECs in confluent monolayers after 40 h. **a**. static conditions. **b**. 4 dyne cm^-2^. **c**. 12 dyne cm^-2^. The dhBMEC nuclei maintain their oval shape under all conditions.


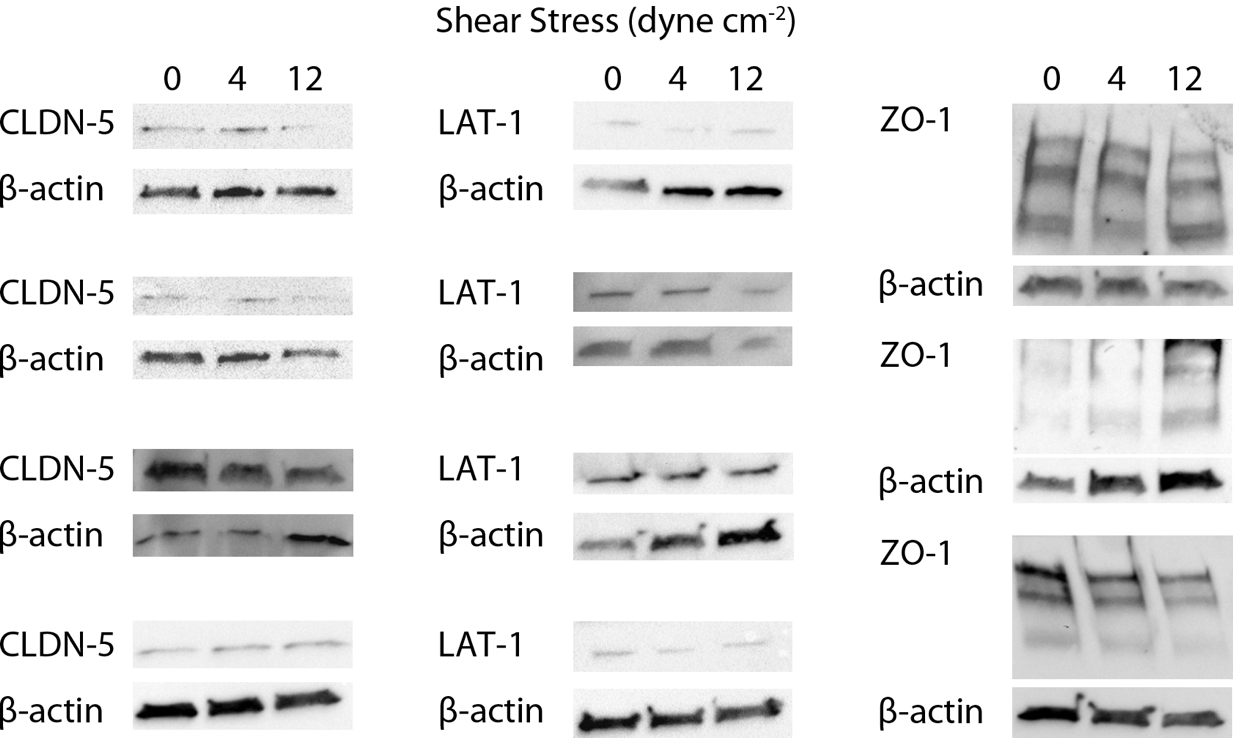


**Figure S7. Western Blots.** Protein expression of CLDN-5, LAT-1, and ZO-1 using western blot. Data were obtained from analysis of four different differentiations for CLDN-5 and LAT-1 and three differentiations for ZO-1. Analysis of the relative intensities of the bands was performed using ImageJ. Each lane was normalized to the intensity of the 0 dyne cm^-2^ lane (see Figure 9a).

**
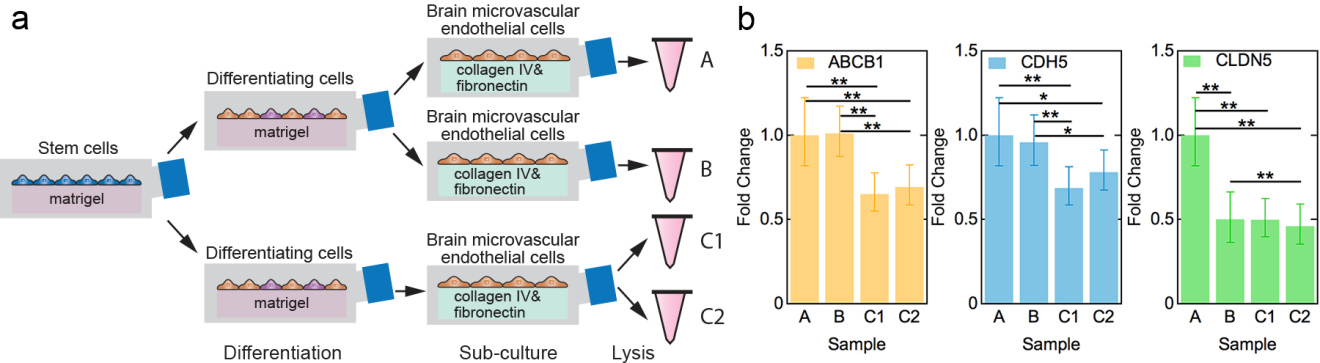
**

**Figure S8. Gene expression variability in the dhBMEC differentiation protocol and qPCR preparation process. a.** Schematic illustration of the steps in the differentiation and qPCR preparation process and samples tested. **b.** Gene expression of ABCB1 (P-gp), CDH5 (VE-cad), and CLDN5 (claudin-5), among samples at various steps in the differentiation/PCR preparation process. Fold changes are reported with respect to Sample A. Error bars represent mean ± SE. GAPDH was used as the housekeeping gene. Since some genes had very high variability between differentiations, we performed qPCR at different steps in the differentiation process to assess any systematic variations. Specifically, we compared variation between differentiations and variation after subculture. We started with stem cells from the same passage split into two flasks. These cells were differentiated at the same time using the same protocol. After 7 days, flask 1 was sub-cultured into two flasks and cultured for 2 days before each flask underwent the lysis procedure, yielding samples A and B. The second flask was sub-cultured into a single flask (at the same concentration as the first flask) for 2 days and lysed. The lysate was separated into two samples (C1 and C2) and the generation of cDNA from the lysates samples were completed separately. Sample A and B were separated from samples C1 and C2 during the differentiation step. Samples A and B were separated from each other at the sub-culture step, whereas samples C1 and C2 were separated during the lysis/cDNA generation step to test which steps in the differentiation and qPCR preparation process contribute to the variability. For this study, we selected three genes: ABCB1 (P-gp), CDH5 (VE-cad), and CLDN5 (claudin-5). The samples that were derived from the same differentiation (A and B; C1 and C2) have similar levels of ABCB1 and CDH5. Samples C1 and C2 have decreased expression of ABCB1 and CDH5 compared to Samples A and B. This indicates that within a differentiation, the ABCB1 and CDH5 levels remain consistent, but there is differentiation-to-differentiation batch variability. CLDN5 levels vary between samples A and B, which suggests the variation here stems from the subculture step. This is the step where the endothelial cells are selected based on adherence to the coating. It is during this step that the endothelial cells form tight junctions with each other, which correlate with the variation in CLDN5 levels, a tight junction protein.
